# The enantiomers of tramadol and its major metabolite inhibit peristalsis in the guinea pig small intestine via differential mechanisms

**DOI:** 10.1186/1471-2210-7-5

**Published:** 2007-03-16

**Authors:** Michael K Herbert, Rebecca Weis, Peter Holzer

**Affiliations:** 1 Department of Anesthesiology, University of Wuerzburg, Wuerzburg, Germany; 2 University of Wuerzburg, Wuerzburg, Germany; 3 Research Unit of Translational Neurogastroenterology, Department of Experimental and Clinical Pharmacology, Medical University of Graz, Graz, Austria

## Abstract

**Background:**

Inhibition of intestinal peristalsis is a major side effect of opioid analgesics. Although tramadol is an opioid-like analgesic, its effect on gut motility is little known. Therefore, the effect of (+)-tramadol, (-)-tramadol and the major metabolite *O*-desmethyltramadol on intestinal peristalsis *in vitro *and their mechanisms of action were examined. Distension-induced peristalsis was recorded in fluid-perfused segments of the guinea pig small intestine. The intraluminal peristaltic pressure threshold (PPT) was used to quantify the motor effects of extraserosally administered drugs.

**Results:**

Racemic tramadol, its (+)- and (-)-enantiomers and the major metabolite *O*-desmethyltramadol (0.1 – 100 μM) concentration-dependently increased PPT until peristalsis was transiently or persistently abolished. The rank order of potency was (-)-tramadol < (+)-tramadol <*O*-desmethyltramadol. The peristaltic motor inhibition caused by (+)- and (-)-tramadol was markedly and that of *O*-desmethyltramadol nearly completely prevented by naloxone, but left unaltered by the 5-hydroxytryptamine receptor antagonists methysergide plus tropisetron. The adrenoceptor antagonists prazosin plus yohimbine reduced the effect of (+)- and (-)-tramadol but not that of *O*-desmethyltramadol.

**Conclusion:**

The results show that the metabolite *O*-desmethyltramadol is more potent in inhibiting peristalsis than its parent compound. The action of all tramadol forms depends on opioid receptors, and that of (+)- and (-)-tramadol also involves adrenoceptors.

## Background

Potent analgesics such as the opioids are required to treat moderate to severe acute and chronic pain. The use of opioids is accompanied by serious side effects such as inhibition of gastrointestinal motility [1]. As a result, several strategies to prevent opioid bowel dysfunction have been envisaged. In particular, opioid-like compounds with a reduced impact on intestinal function and opioid receptor antagonists with a peripherally restricted site of action have been developed [2,3]. Tramadol is a centrally acting synthetic 4-phenylpiperidine analogue of codeine, which has become available as an analgesic for the treatment of moderate to severe pain [4]. Its efficacy has been confirmed in postoperative [5,6], neuropathic [7,8] and osteoarthritic pain [9,10] as well as in patients suffering from chronic pancreatitis [11]. The analgesic potency of tramadol appears to depend on the pain state under treatment and has been described as similar [12] or, in the case of pancreatitis, even superior to that of morphine [11]. However, there is controversial information as to whether tramadol has an inhibitory action on gut motility in humans, and there is little known as to which mechanisms underlie such an effect [9,12].

The analgesic effect of tramadol is mediated through two distinct but complementary mechanisms of action. It acts as an opioid agonist with selectivity for the μ-opioid receptor and binds weakly to the κ- and δ-opioid receptor [13]. Tramadol is extensively metabolised in the liver, and the *O*-desmethyl metabolite displays a 200-fold higher affinity for opioid receptors than the parent drug [4]. The analgesic and antinociceptive effects of tramadol are only partially antagonised by the opioid antagonist naloxone, which suggests that nonopioid mechanisms are also involved [4,13]. Thus, at the same concentrations at which it binds to opioid receptors, tramadol acts on monoamine systems to inhibit the reuptake of norepinephrine and serotonin [13-15]. Furthermore, the (+)- and (-)-enantiomers differentially contribute to the analgesic effect of racemic tramadol which is the clinically used form of the drug. The (+)-enantiomer has a higher affinity for the μ-receptor and is a more effective inhibitor of 5-HT reuptake, whereas the (-)-enantiomer is a more effective inhibitor of norepinephrine reuptake and increases norepinephrine release by autoreceptor activation.

In view of these findings the current study pursued three aims. The first goal was to explore whether tramadol is able to inhibit intestinal peristalsis and to characterize this action in quantitative terms. Since tramadol is available in the form of the (+) and (-) enantiomer and the compound is metabolised to the active metabolite *O*-desmethyltramadol [16], the second aim was to compare (+)- and (-)-tramadol as well as *O*-desmethyltramadol in their potency and efficacy to depress intestinal peristalsis. The third aim of the study was to explore whether any effect of tramadol on intestinal peristalsis is exclusively due to activation of opioid receptors or whether there is also an opioid receptor-independent component of action. Further experiments were designed to differentiate between an involvement of 5-hydroxytryptamine (5-HT) receptors and adrenoceptors in the opioid receptor-independent inhibition of peristalsis due to tramadol.

## Results

**Figure 1:**
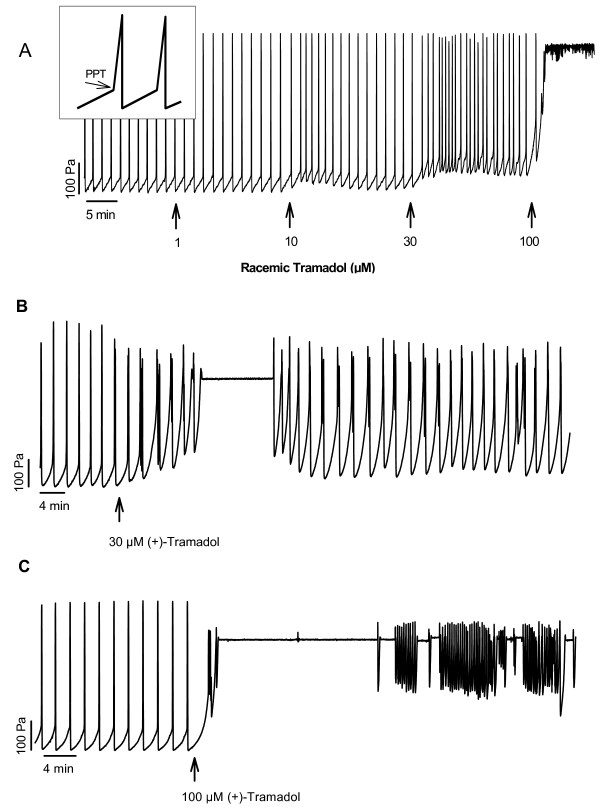
**Original recordings of the effect of racemic and (+)-tramadol on peristalsis**. (A) Luminal perfusion of isolated segments of the guinea pig small intestine slowly increased the intraluminal pressure; when the peristaltic pressure threshold (PPT, marked by an arrow in the inset of A was reached, a peristaltic wave shown as a spike-like increase of intraluminal pressure was triggered. Increasing concentrations of racemic tramadol were cumulatively added to the bath. Peristaltic activity was constant during a control period and remained uninfluenced by 1 μM racemic tramadol. PPT was concentration-dependently increased by 10 and 30 μM racemic tramadol and peristalsis was completely abolished by 100 μM of the drug. (B) The concentration of 30 μM (+)-tramadol added singly to the bath caused an increase in PPT and peristalsis ceased for about 10 min. Thereafter peristaltic contractions returned, however, with a markedly higher PPT. (C) Peristaltic contractions were completely abolished by 100 μM (+)-tramadol added singly to the bath, so that despite an intraluminal pressure of 400 Pa no peristalsis took place. The high-frequency oscillations of intraluminal pressure in the later part of the tracing represent uncoordinated gut wall motions without any propulsion of the intraluminal contents.

**Figure 2:**
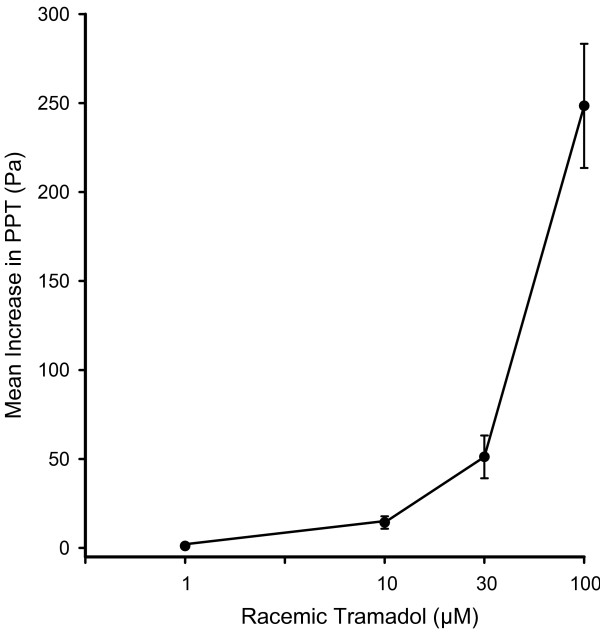
**Concentration-response relationship for the increase in PPT (Pa) caused by racemic tramadol (1–100 μM)**. The symbols represent the mean values ± SEM of PPT recorded from 6 intestinal segments.

Under control conditions regular peristaltic contractions were recorded that stayed constant in all experiments and were not influenced by the addition of vehicle (Tyrode's solution, data not shown). A pilot study showed that racemic tramadol (1–10–30–100 μM) administered to the organ bath in a cumulative manner increased PPT in a concentration-dependent fashion (Figs 1A and 2). Peristalsis was totally abolished by 100 μM racemic tramadol (Fig. 1A) in 5 of 6 segments.

**Figure 3:**
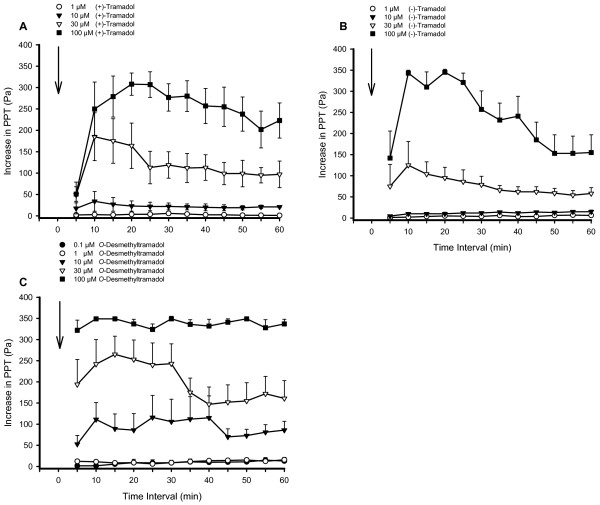
**Concentration- and time-related effect of various forms of tramadol to increase PPT**. (A) (+)-tramadol (1 – 100 μM), (B) (-)-tramadol (1 – 100 μM), and (C) *O*-desmethyltramadol (0.1–100 μM) increased PPT in a concentration dependent manner. The time of administration of (+)-tramadol, (-)-tramadol and *O*-desmethyltramadol is indicated by an arrow. The symbols represent the mean values of PPT recorded from 6 segments at consecutive 5 min intervals; the positive error bars show the standard error of the mean (SEM).

Addition of (+)- and (-)-tramadol to the organ bath led to an increase of PPT and compromised ileal peristaltic contractions in a concentration-related manner. While Tyrode's solution as well as 1 μM (+)-tramadol and (-)-tramadol did not affect peristalsis, PPT was transiently enhanced by 10 μM (+)-tramadol (Fig. 1B, 3A). A more pronounced increase of PPT was caused by 30 μM (+)- and (-)-tramadol (mean Δ PPT 118.8 ± 31.3 Pa and 77.1 ± 20.9 Pa, respectively). An increase in the frequency of peristaltic waves was also observed. Peristalsis completely ceased with a latency of 8.3 ± 4.1 min and a duration of 9.7 ± 0.4 min in two segments exposed to 30 μM (+)-tramadol. Regular peristalsis reoccurred in these two segments, however, with a slightly higher PPT than in the initial control period. Administration of 100 μM (+)- and (-)-tramadol abolished peristalsis after 11.2 ± 4.3 min and 5.3 ± 0.8 min, respectively, in all segments tested (Fig. 1C). In 5 of 6 segments inhibited by 100 μM (-)-tramadol spontaneous ileal contractions recovered 9.2 ± 3.0 min with intermittend periods of disturbed motility. The mean increase of PPT due to 100 μM (+)-tramadol was more prolonged than that due to 100 μM (-)-tramadol (Figs 3A and 3B).

*O*-Desmethyltramadol had a stronger inhibitory effect on peristalsis than the (+)- and (-)-tramadol enantiomers. The lowest concentrations of 0.1 and 1 μM *O*-desmethyltramadol did not affect the PPT, but peristalsis was already inhibited by 10 μM *O*-desmethyltramadol for 18.3 min in one segment. The mean increase of PPT due to 10 μM *O*-desmethyltramadol during the observation period of 60 min was 91.8 ± 32.9 Pa (Fig. 3C). At 30 μM, *O*-desmethyltramadol abolished peristalsis transiently in 3 of 6 segments after a latency of 3.6 ± 1 min and on average increased PPT by 239.1 ± 51.9 Pa in all segments tested during the first 20 min post-administration (Fig. 3C). Peristalsis was completely blocked by 100 μM *O*-desmethyltramadol in all segments, an effect that took place in less than 4 min after addition of the drug. Inhibition of peristalsis manifested itself either as complete absence of any motor activity or as stationary high-frequency oscillations of intraluminal pressure, which did not propel the intraluminal contents.

**Figure 4:**
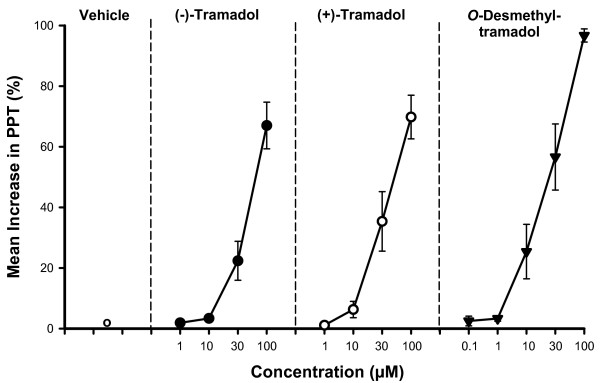
**Concentration-response relationships for the increase in PPT (Pa) caused by various forms of tramadol**. The concentration-response relationships for the increase in PPT (Pa) caused by (-)-tramadol, (+)-tramadol (1–100 μM) and *O*-desmethyltramadol (0.1–100 μM) is compared to that seen after vehicle (Tyrode's solution) administration. The symbols represent means with SEM, n = 6 segments.

The inhibitory potency of (-)-tramadol to impair intestinal peristalsis (EC_50 _= 73 μM) was lower than that of (+)-tramadol (EC_50 _= 53 μM), and the metabolite *O*-desmethyltramadol with an EC_50 _= 13.6 μM was more potent than either enantiomer (Fig. 4).

**Figure 5:**
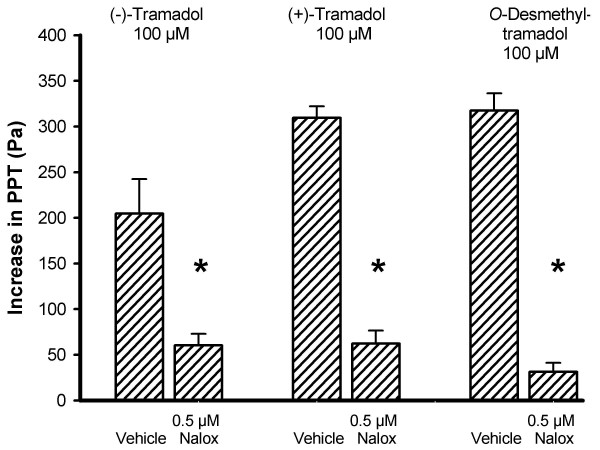
**Naloxone (Nalox) attenuates the inhibitory effect of tramadol on peristalsis**. The increase in PPT due to 100 μM (-)-tramadol, (+)-tramadol and *O*-desmethyltramadol was significantly reduced by pre-treatment with the pan-opioid receptor antagonist naloxone (0.5 μM). The bars represent means with SEM, n = 6 segments, *P < 0.05 as compared with vehicle (Tyrode's solution).

Pre-treatment with the pan-opioid receptor antagonist naloxone (0.5 μM) prevented the inhibition of intestinal peristalsis caused by tramadol. The reduction of the maximum inhibitory effect of 100 μM (-)-tramadol, 100 μM (+)-tramadol and 100 μM *O*-desmethyltramadol on PPT was 70 %, 80 % and 90 %, respectively (Fig. 5).

**Figure 6:**
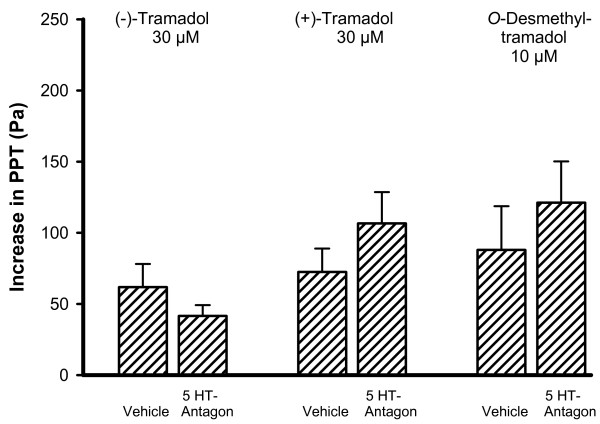
**The inhibitory effect of tramadol on peristalsis remained unaffected by 5-HT receptor antagonists (5-HT Antagon)**. The increase in PPT due to the submaximally effective concentrations of 30 μM (-)-tramadol, (+)-tramadol and *O*-desmethyltramadol was not reduced by pre-treatment with the combination of the 5-HT receptor antagonists methysergide (1 μM) plus tropisetron (1 μM). The bars represent means with SEM, n = 6 – 8 segments. There was no significant difference relative to the values measured after vehicle (Tyrode's solution) administration.

**Figure 7:**
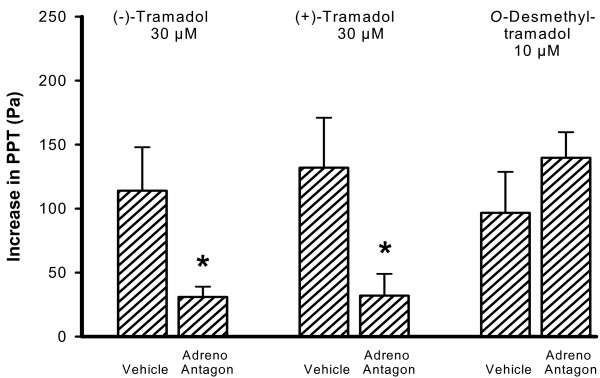
**Inhibitory effect of tramadol on peristalsis after pre-treatment with adrenoceptor antagonists (Adreno Antagon)**. The increase in PPT due to the submaximally effective concentrations of 30 μM (-)-tramadol and 30 μM (+)-tramadol was significantly reduced by the combination of prazosin (α_1_-adrenoceptor antagonist, 1 μM) plus yohimbine (α_2_-adrenoceptor antagonist, 1 μM), whereas the effect of 10 μM *O*-desmethyltramadol on PPT was not affected. The bars represent means with SEM, n = 6 segments. *P < 0.05 as compared with vehicle (Tyrode's solution).

Pre-treatment with a combination of the 5-HT receptor antagonists methysergide plus tropisetron did not affect the increase of PPT caused by the equieffective concentrations of 30 μM (-)-tramadol, 30 μM (+)-tramadol and 10 μM *O*-desmethyltramadol (Fig. 6). In contrast, the inhibitory effect of 30 μM (+)-tramadol and 30 μM (-)-tramadol was significantly reduced by the combination of the α_1_- and α_2_- adrenoceptor antagonists prazosin and yohimbine (Fig. 7), while the PPT increase due to 10 μM *O*-desmethyltramadol remained unchanged.

As summarized in Table 1, the receptor antagonists used here caused only minimal changes of PPT by themselves. PPT slightly decreased due to naloxone and slightly increased due to the 5-HT receptor antagonists, with mean PPT changes lower than 15 Pa.

**Table 1:** Change of PPT due to Vehicle and Various Antagonists during a Period of 20 min

		Δ PPT (Pa)
Vehicle (Tyrode's solution)	n = 54	0.31 ± 1.9
Naloxone (0.5 μM)	n = 18	-10.4 ± 1.6
Methysergide (1 μM) + tropisetron (1 μM)	n = 18	13.4 ± 2.5
Yohimbine (1 μM) + prazosin (1 μM)	n = 18	3.0 ± 2.0

Data represent means ± SEM

## Discussion

The (+)- and (-)-enantiomers of tramadol and its major metabolite *O*-desmethyltramadol impaired intestinal peristalsis in a concentration-dependent manner as deduced from an increase in the peristaltic pressure threshold. The increase in the frequency of the peristaltic waves which was also seen following exposure to submaximal concentrations of tramadol is likely to reflect an impairment of the peristaltic contractions resulting in incomplete emptying of the intestinal segments. *O*-desmethyltramadol was more potent than the parent compounds (+)- and (-)-tramadol. Overall the inhibitory potency of (+)-tramadol, (-)-tramadol and *O*-desmethyltramadol was considerably lower than that of the classical opioids such as fentanyl, sufentanil and morphine, which have an EC_50 _in the nanomolar range as tested in the same experimental *in vitro *setup [18,20]. Codeine, the parent molecule of tramadol has an EC_50 _of 21 μM, which is in the same order of magnitude as *O*-desmethyltramadol (EC_50 _= 13.5 μM) [18]. The stronger inhibitory effect on gut motility of *O*-desmethyltramadol is in line with its more potent analgesic capacity compared to that of both enantiomers [4].

The current data are of clinical relevance, given that the ability of tramadol to inhibit gastrointestinal motility in humans is controversial. In one study tramadol was found to slow colonic transit time to a small extent in healthy volunteers, but had no effect on upper gastrointestinal motility [21]. Another study found tramadol to have a measurable but smaller inhibitory effect on gastric emptying than morphine and codeine [22]. In patients suffering from chronic pancreatitis, orocecal transit time remained unchanged after 5 days of tramadol treatment, but increased with morphine [11]. Gastrointestinal motility was also assessed after abdominal hysterectomies in patients receiving 48-h infusions of morphine or tramadol postoperatively in a randomized and double-blind fashion [12]. Orocecal and colonic transit times increased after infusion of both morphine and tramadol, but gastric emptying was prolonged only with morphine. In randomized and placebo-controlled studies of patients with diabetic neuropathy and polyneuropathy, who received 210 mg or 200–400 mg tramadol per day with good analgesic benefit, a constipating effect of tramadol was observed [7,8]. Studies in patients suffering from cancer have shown that tramadol has a constipating effect, which, however, is less severe than that of morphine [23]. The present results show that tramadol has the potential to inhibit peristalsis in a concentration-dependent manner.

Another aim of this study was to explore the mechanisms underlying the inhibitory action of the tramadol enantiomers and *O*-desmethyltramadol on intestinal peristalsis. The increase of PPT due to tramadol could to a large extent be prevented by the opioid receptor antagonist naloxone, and the inhibition due to *O*-desmethyltramadol was in fact attenuated by 90 %. This confirms that most of the inhibitory effect of tramadol on gut motility depends on opioid receptor activation, whereas its analgesic effect could be antagonized with naloxone by 30 % only [24]. A major part of the tramadol-induced analgesia is mediated by reuptake inhibition and release of serotonin and norepinephrine [13-15]. Of these two transmitters, only norepinephrine seems to play an appreciable role in the inhibitory action of (+)- and (-)-tramadol on peristalsis, since the combination of the α_1_- and α_2_- adrenoceptor antagonists prazosin plus yohimbine diminished the inhibitory effect of both enantiomers to a significant extent. The localization of these adrenoceptors to the various components of the peristaltic nerve-muscle circuitry underlying peristalsis awaits to be addressed. In contrast, peristaltic motor inhibition due to the metabolite *O*-desmethyltramadol remained unchanged by α_1_- and α_2_- adrenoceptor antagonism. The 5-HT receptor antagonists methysergide plus tropisetron (antagonists at 5-HT_1_, 5-HT_2 _and 5-HT_7 _as well as 5-HT_3 _receptors) did not significantly affect the motor action of (+)-tramadol, (-)-tramadol and *O*-desmethyltramadol. It was beyond the scope of this study to analyze the possible implication of all 5-HT receptor subtypes present in the gastrointestinal tract. In agreement with its analgesic action, the inhibition of peristalsis due to *O*-desmethyltramadol is more pronounced than that due to (+)- and (-)-tramadol and the inhibitory motor action of *O*-desmethyltramadol appears to be solely mediated by opioid receptor mechanisms.

The present data raise the important question as to how the concentrations of (+)-tramadol, (-)-tramadol and *O*-desmethyltramadol found to inhibit peristalsis in the *in vitro *setting compared with the concentrations of tramadol, its enantiomers and metabolite that are analgesic under clinical conditions *in vivo*. In this respect, the evaluation of drug effects on *in vitro *organ bath preparations has both advantages and limitations. *In vitro*, environmental conditions such as temperature, oxygenation, composition of the bath solution and drug concentration can be kept constant in a controlled manner. However, protein binding, distribution, metabolism and excretion of drugs cannot be modelled *in vitro*. As a consequence, the pharmacokinetic and pharmacodynamic conditions of drug action differ considerably between the *in vitro *and *in vivo *settings. In this context it is evident that our *in vitro *preparation has the advantages (i) to reveal effects of low drug concentrations on peristalsis, (ii) to describe these effects in quantitative terms, (iii) to confirm that the observed drug effects arise exclusively from interaction with intestinal structures and mechanisms and (iv) to reveal that the observed drug effects are independent of changes in local, regional and systemic perfusion and other hemodynamic parameters.

In systematic pharmacokinetic studies, plasma concentrations of tramadol, its enantiomers and *O*-desmethyltramadol have been found to show considerable interindividual variations [25-28]. Depending on the study design the minimum effective concentration of tramadol to cause analgesia is in the range of 0.1 – 10 μM [25,28]. Compared with the concentrations that inhibited intestinal peristalsis in the current study, these figures suggest that clinically relevant plasma concentrations of tramadol, its enantiomers and metabolite will gradually impair intestinal peristalsis in a concentration-dependent manner. However, the concentrations of (+)-tramadol, (-)-tramadol and *O*-desmethyltramadol (100 μM) that completely block peristalsis are unlikely to be reached under routine clinical conditions.

## Conclusion

Racemic tramadol, its (+) and (-) enantiomers as well as their major metabolite *O*-desmethyltramadol inhibit peristalsis in the guinea pig small intestine at micromolar concentrations. Since the clinically relevant plasma concentrations of tramadol are below those that abolish peristalsis in the guinea pig small bowel, it is tentatively concluded that tramadol is an alternative to classical opiate therapy as it has a more favourable profile of gastrointestinal adverse effects.

## Methods

### Animals

After obtaining approval from the Animal Care and Use Committee at the Government of Lower Franconia, Wuerzburg, Germany, adult guinea-pigs (BFA strain, Charles-River Wiga, Sulzfeld, Germany) of either sex weighing between 340 and 470 g were stunned and bled via the carotid arteries. The guinea pigs were not used before 3 days after their arrival at the animal care facility.

They were housed in standard cages within the departmental animal house and received standard guinea-pig food and water ad libitum until the time of the experiment.

### Experimental and recording protocol

The experimental and recording protocol has been described in detail [17]. In brief, the jejunum and ileum were excised, flushed of luminal contents and placed in Tyrode's solution at room temperature, gassed with 95% O_2 _and 5% CO_2_, until used. For studying peristalsis, the distal small intestine (at least 10 cm proximal to the ileocecal valve) was divided into segments, each being approximately 8 to 10 cm long. Five intestinal segments were set up in parallel in silanized glass organ baths containing 30 mL of Tyrode's solution at 37°C. The oral end of the intestinal segment was tied to an inflow cannula, which permitted the continuous infusion of prewarmed Tyrode's solution at a flow rate of 0.5 mL min^-1^. The aboral end of the segment was attached to an intermediate tubing fixed with a T piece. One arm of the T piece was connected to a pressure transducer for recording of the intraluminal pressure and the other arm of the T piece was fitted with a vertical outlet tubing that ended 4 cm above the fluid level of the organ bath. This arrangement made emptying of the intestinal segment possible when peristaltic contractions raised the intraluminal pressure above 400 Pascal (Pa). When the intraluminal pressure reached a threshold (peristaltic pressure threshold, PPT, inset of Fig. 1A), an aborally moving wave of peristaltic contraction was triggered and the emptying phase of peristalsis initiated. The wave of circular muscle contraction, measured as a spike-like increase in intraluminal pressure, propelled the intraluminal fluid to leave the system via the outlet tubing and thus caused emptying of the segment. The intraluminal pressure at the aboral end of the segments was measured with a pressure transducer whose signal was, via an analog/digital converter, fed into a personal computer and recorded simultaneously on a pen-recorder.

The preparations were allowed to equilibrate in the organ bath for a period of 30 min. Thereafter the bath fluid was renewed and peristaltic motility initiated by intraluminal perfusion of the segments. After basal peristaltic activity had been recorded for at least 30 min, the drugs to be tested were administered to the bath, i.e., to the serosal surface of the intestinal segments. All vehicle solutions used in this study were tested separately to ensure that they were devoid of any influence on peristaltic activity.

Pilot experiments were designed to explore the overall action of racemic tramadol on intestinal peristalsis. For this purpose, six ileal segments from six different guinea pigs were exposed to racemic tramadol which was added to the organ bath in a cumulative manner at concentrations of 1–10–30–100 μM in intervals of 20 min.

In separate series of experiments (i-iii) the effects of the two enantiomers (+)- and (-)-tramadol and of the major metabolite *O*-desmethyltramadol on gut motility were investigated in a more detailed manner. Each concentration of (+)-tramadol, (-)-tramadol (1,10,30,100 μM) and *O*-desmethyltramadol (0.1,1,10,30,100 μM) was tested on six segments from six different guinea pigs and each segment was exposed to only one drug concentration. The peristaltic contractions were recorded for 60 min after addition of the drug. To explore whether (+)-tramadol, (-)-tramadol and *O*-desmethyltramadol exert their inhibitory action on gut motility through (iv) activation of opioid receptors or interference with the reuptake of (v) 5-HT and (vi) norepinephrine the following experiments were performed: (iv) Naloxone (0.5 μM) was given into the organ bath 20 min prior to the addition of 100 μM (+)-tramadol, (-)-tramadol and *O*-desmethyltramadol. The combination of (v) 1 μM methysergide (nonselective antagonist at 5-HT receptors, predominantly 5-HT_1_, 5-HT_2_, 5-HT_7_) plus 1 μM tropisetron (selective antagonist at 5-HT_3 _receptors) or of (vi) 1 μM prazosin (antagonist at α_1_-adrenoceptors) plus 1 μM yohimbine (antagonist at α_2_-adrenoceptors) was added to the organ bath 20 min prior to administration of the submaximally effective concentrations of 30 μM (+)-tramadol, (-)-tramadol and 10 μM *O*-desmethyltramadol. The effect of tramadol recorded in the presence of the antagonists was compared with that seen in the presence of vehicle (Tyrode's solution).

### Drugs

(+)-Tramadol, (-)-tramadol and *O*-desmethyltramadol were supplied by Gruenenthal (Stollberg, Germany) and tropisetron was purchased from Novartis Pharma Ltd. (Nuernberg, Germany). All other chemicals were from commercial sources and of the highest purity available. The chemicals were dissolved in sterile water, and stock solutions were further diluted with Tyrode's solution before use. The composition of the Tyrode's solution was (in mM): NaCl 136.9, KCl 2.7, CaCl_2 _1.8, MgCl_2 _1.0, NaHCO_3 _11.9 and NaH_2_PO_4 _0.4 and glucose 5.6.

### Calculation of data

PPT was used to quantify drug effects on peristalsis. After regular peristaltic contractions had been recorded for at least 20 min, the PPT of the last peristaltic wave immediately before drug addition was taken as baseline. Following drug administration the PPT of the last complete peristaltic wave within consecutive 5 min periods (i.e., 5, 10, 15 and further up to 60 min post-administration) was calculated. Inhibition of peristalsis was reflected by an increase in PPT, and abolition manifested itself in a lack of propulsive motility in spite of an intraluminal pressure of 400 Pa as set by the position of the outlet tubing. Although in this case PPT exceeded 400 Pa, abolition of peristalsis was expressed quantitatively by assigning PPT a value of 400 Pa in order to obtain numerical results suitable for further statistical evaluation [17,18].

In order to obtain the net increase of PPT caused by (+)-, (-)-tramadol and *O*-desmethyltramadol, the baseline PPT was subtracted from the respective PPT values recorded in the presence of (+)-tramadol, (-)-tramadol and *O*-desmethyltramadol.

### Statistics

The data were analysed for normal distribution using the Kolmogorov-Smirnov-Test. Since normality was confirmed, all quantitative data are presented as mean ± SEM (standard error of the mean). Significance was evaluated using ANOVA and *post hoc *Student-Newman-Keuls or Student *t *test as appropriate for a level of P < 0.05 (Sigma Stat for Windows, version 2.03, SPSS Inc., Chicago, IL). The 50% effective concentration value (EC_50 _value) for (+)-tramadol, (-)-tramadol and *O*-desmethyltramadol was calculated by the method of Tallarida and Murray [19].

## Authors' contributions

MKH and RW contributed equally to this work. RW performed the experiments and drafted the manuscript. MKH and RW designed the studies and analyzed the data. MKH and PH organized all experiments on peristalsis and prepared the manuscript. All authors read and approved the manuscript.
